# Hacking the Lipidome: New Ferroptosis Strategies in Cancer Therapy

**DOI:** 10.3390/biomedicines12030541

**Published:** 2024-02-28

**Authors:** Borys Varynskyi, Joel A. Schick

**Affiliations:** 1 Genetics and Cellular Engineering Group, Research Unit Signaling and Translation, Helmholtz Zentrum Munich, Ingolstädter Landstr. 1, 85764 Neuherberg, Germany; joel.schick@helmholtz-muenchen.de; 2 Physical and Colloidal Chemistry Department, Pharmaceutical Faculty, Zaporizhzhia State Medical and Pharmaceutical University, 26 Maiakovskoho Ave., 69035 Zaporizhzhia, Ukraine

**Keywords:** ferroptosis, cancer cells, de novo lipogenesis, ketolysis, lipidomics, metabolic reprogramming, cancer treatment, therapeutic resistance

## Abstract

The concept of redirecting metabolic pathways in cancer cells for therapeutic purposes has become a prominent theme in recent research. Now, with the advent of ferroptosis, a new chink in the armor has evolved that allows for repurposing of ferroptosis-sensitive lipids in order to trigger cell death. This review presents the historical context of lipidomic and metabolic alterations in cancer cells associated with ferroptosis sensitization. The main proferroptotic genes and pathways are identified as therapeutic targets for increasing susceptibility to ferroptosis. In this review, a particular emphasis is given to pathways in cancer cells such as de novo lipogenesis, which has been described as a potential target for ferroptosis sensitization. Additionally, we propose a connection between ketolysis inhibition and sensitivity to ferroptosis as a new vulnerability in cancer cells. The main proferroptotic genes and pathways have been identified as therapeutic targets for increasing susceptibility to ferroptosis. Proferroptotic metabolic pathways and vulnerable points, along with suggested agonists or antagonists, are also discussed. Finally, general therapeutic strategies for ferroptosis sensitization based on the manipulation of the lipidome in ferroptosis-resistant cancer cell lines are proposed.

## 1. Introduction: Ferroptosis History and Concept

The first report on ferroptosis was published in 2012 [1], marking the emergence of a unique cell death mechanism. The authors of the summary of ten years of ferroptosis demonstrated that its characteristics had been observed for several decades, but it was not until 2012 that it was established as a distinct form of cell death [2]. Providing a historical background is necessary for a better understanding of the development of the concept of ferroptosis and the mechanisms associated with it, based on data collected over the last decade.

**Figure 1:**
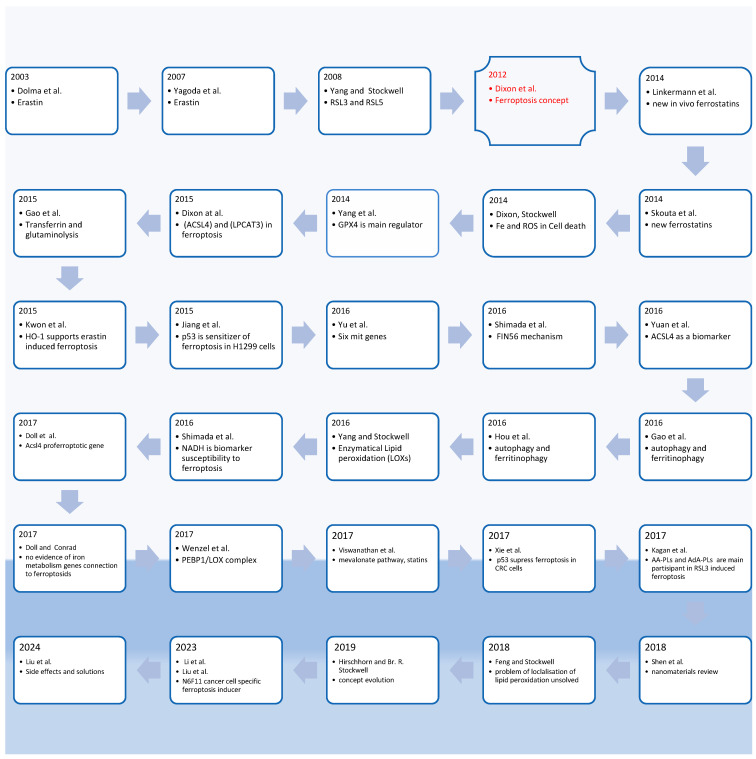
Illustration of the history of ferroptosis based on analysis conducted using the “Connected papers” tool [1,2,4,5,6,7,8,9,10,11,12,13,14,15,16,17,18,19,20,21,22,23,24,25,26,27,28,29,30,31,32].

As a result, ferroptosis has a research history spanning 12 years (Figure 1), with the initial publication on the mechanism appearing nine years after the discovery of its inducers. Ferroptosis research encompasses a vast number of papers, and a cursory historical analysis of the literature revealed the most salient papers related to ferroptosis (Figure 1) [3].

The discovery of erastin marked the beginning of ferroptosis research [4,5], followed by the discovery of RSL3 and RSL5 [6].

Subsequently, numerous scientists became involved in studying the phenomena of ferroptosis. Linkermann et al. reported that ferrostatin-1, an inhibitor of ferroptosis, displayed poor stability in vivo and proposed a new generation of ferrostatins, such as SRS16-86, which demonstrated efficacy in vivo [7]. Another article described novel ferrostatins, with SRS11-92 being identified as the most sensitive [8]. The authors conducted an in-depth analysis of iron and reactive oxygen species (ROS)-dependent mechanisms of cell death [9]. They found that *GPX4* knockdown rendered cancer cells highly susceptible to RSL3-induced ferroptosis, while its overexpression conferred resistance [10].

Dixon et al. suggested that the activity of the *ACSL4* (Acyl-CoA Synthetase Long Chain Family Member 4) and *LPCAT3* (Lysophosphatidylcholine Acyltransferase 3) genes demonstrated sensitivity to ferroptosis [11]. Glutaminolysis and transferrin were identified as essential factors for ferroptosis [12]. Kwon et al. proposed that ferroptosis was supported by iron-containing heme oxygenase-1 (*HO-1*) [13]. Activation of *p53* sensitized H1299 cells led to erastin-induced ferroptosis through increased ROS production without DNA damage [14]. The mitochondria-related genes *RPL8*, *IREB2*, *ATP5G3*, *CS*, *TTC35*, and *ACSF* were found to regulate ferroptosis, and the authors reviewed cancer cells that were sensitive to ferroptosis [15]. Shimada et al. showed using the example of FIN56 that a third type of ferroptosis inducers exist and are destructors of GPX4 [16]. *ACSL4* was found to be more highly expressed in ferroptosis-sensitive cell lines (HepG2 and HL60) compared to resistant cancer cells (LNCaP and K562) [17]. Ferritinophagy, associated with *NCOA4*, was identified as a participant in ferroptosis due to an increase in the labile iron pool (LIP) [18]. Hou et al. also confirmed the role of ferritin autophagy (genes *Atg5* and *Atg7*) in erastin-induced ferroptosis, showing that *NCOA4* knockdown led to a decrease in Ferritinophagy and blocked ferroptosis [19]. Yang and Stockwell proposed that lipid peroxidation in ferroptosis is a controlled process involving iron-containing enzymes, with potential involvement of lipoxygenases [20]. Shimada et al. discovered that NADPH serves as a biomarker of susceptibility to ferroptosis, either due to increased NADPH concentrations (low [NADP+]/[NADPH]) or because stability to NADPH elimination by FIN56 corresponds to resistant cell lines [21]. Doll et al. confirmed, through *ACSL4* knockout experiments, that the *ACSL4* gene is essential for ferroptosis sensitization as it activates the inclusion of arachidonate (AA) into the membrane phospholipids [22]. 

A review [23] focused on unresolved issues regarding the mechanisms of ferroptosis, examining the perceived necessity of LOXs in the process. For example, the review looked into why LOX knockdowns have shown ferroptosis inhibition in erastin-induced models, but not in RSL3 induction. Additionally, the role of iron metabolism-regulated genes in ferroptosis remains unclear. Another question raised pertains to why omega-6 polyunsaturated fatty acids (PUFAs) sensitize *ACSL4* knockout cells to ferroptosis, whereas omega-3 PUFAs do not.

Wenzel et al. revealed the role of the phosphatidylethanolamine-binding protein 1/15-lipoxygenase (PEBP1/15LOX) complex in ferroptosis. They found that 15LOX, when not bound to PEBP1, peroxidizes free fatty acids but has minimal effect on PUFA-PLs. However, the PEBP1/15LOX complex is capable of oxidizing PUFA-PLs [24].

In another study [25], it was shown that inhibitors of 3-hydroxy-3-methylglutaryl coenzyme A reductase (HMG-CoA reductase), known as statins, interrupt the synthesis of GPX4 by inhibiting the synthesis of isopentenyl pyrophosphate in the cholesterol biosynthesis pathway. This inhibition helps induce ferroptosis in cells involved in epithelial–mesenchymal transition (EMT). Importantly, the influence of statins cannot be reversed by lipophilic antioxidants.

In CRC cells (HCT116 and SW48), p53 suppresses ferroptosis by blocking DPP4 activity and preventing DPP4-NOX binding. Inhibition or knockout of *TP53* in CRC cells makes them susceptible to ferroptosis induced by erastin [26]. Kagan et al. suggest that AA-PE (arachidonic acid—phosphotidylethanolamines) and AdA-PE (adrenic acid—phosphotidylethanolamines) are responsible for lipid peroxidation during ferroptosis induced by RSL3 [27].

Shen et al. [28] provided a review of modern strategies for cancer ferroptotic therapy. They discussed clinically approved ferroptosis inducers such as sorafenib, sulfasalazine, artemisinin, artesunate, and dehydroartemisinin. The authors also described the application of nanomaterials for inducing ferroptosis therapy.

H. Feng and Br. R. Stockwell discussed possible areas of localization for lipid peroxidation: membranes, mitochondria, ER (endoplasmic reticulum), and lysosomes. Localization, as well as the essentiality of the involvement of different organelles and their membrane compartments, is not completely resolved [29]. 

Hirschhorn and Stockwell described the evolution of ferroptosis concept in focus on positive and negative regulation, ferroptosis related diseases, and its relation to cancer. The concept that it is programmed, rather than accidental, cell death was underscored [2]. 

Li et al. and Liu et al. discovered a new specific ferroptosis inducer called N6F11, which selectively attacks cancer cells while sparing immune cells [30,31]. 

The authors discussed the side effects associated with classical ferroptosis inducers, highlighting their low selectivity for cancer cells and potential harm to normal cells. They supported the discovery of N6F11 as a promising alternative [32].

In summary, the concept of ferroptosis can be defined as an iron-dependent, non-apoptotic, non-necrotic form of regulated cell death. It is characterized by specific morphological changes and is regulated by alternative genetic, protein, metabolic, and execution mechanisms. Ferroptosis is associated with increased levels of lipid reactive oxygen species (ROS) when the systems responsible for glutathione (GSH)-dependent lipid peroxide recovery are compromised. Ferroptosis can be prevented by lipophilic antioxidants, iron chelators, inhibitors of lipid peroxidation, and depletion of polyunsaturated fatty acyl phospholipids (PUFA-PLs) that are susceptible to ROS-induced damage [2,27].

Ferroptosis is a form of regulated cell death (RCD) that is primarily driven by lipid peroxidation and does not involve caspases. Intrinsic apoptosis is a type of RCD characterized by mitochondrial outer membrane permeabilization and participation of CASP3 and dependent on the inner cell changes. Extrinsic apoptosis, on the other hand, is initiated through death receptors and involves the activation of CASP8 and CASP3, triggered by external factors influencing the cell’s outer surface [33].

### Key Points in Ferroptosis Historical Observation and Purpose of This Review

Reactive oxygen species (ROS) include oxygen ions, oxygen-containing free radicals, and peroxides. ROS peroxidate lipids through a free radical chain mechanism.

Indicators of ferroptosis include the inhibition of GPX4′s ability to recover phospholipids (PL) after lipid peroxidation, the presence of redox-active iron, and the peroxidation of PUFA-PLs [34].

The topic of ferroptosis is of great importance due to its involvement in the pathophysiology of various degenerative diseases, including cardiovascular diseases, digestive system and liver diseases, neurological disorders, urinary and reproductive system diseases, and immune system disorders [35,36]. Ferroptosis also holds promise as a potential approach for cancer treatment, especially for drug-resistant cancer types [37]. 

Several paradoxes surrounding ferroptosis have been partially or completely resolved over the past decade. One such question concerns the mechanism of lipid peroxidation in ferroptosis—is it genetically determined, controlled by enzymatic processes, or a chaotic free radical chain process?

A review on this topic is necessary to summarize the data on the relationship between the lipid and metabolite composition of cells and their sensitivity to ferroptosis. A better understanding of lipid compositions in cancer cells can help identify new strategies to sensitize them to ferroptosis.

The purpose of this review is to provide a comprehensive understanding of the concept of ferroptosis, with a focus on identifying specific lipid target pathways that can sensitize cancer cells while sparing normal cells. The review aims to explore the vulnerabilities in the lipidome of ferroptosis-resistant cancer cells and propose potential strategies to overcome them. Additionally, the review will address the existing ambiguities and contradictions in the literature regarding the concept of ferroptosis. It will strive to provide possible solutions or explanations for these inconsistencies and highlight the prospect of utilizing ferroptosis as a therapeutic approach for various diseases.

## 2. Cancer Cells Metabolome and Lipidome Alteration

The origin of cancer has been primarily attributed to somatic cell mutations [38,39]. It is widely known that these mutations accumulate with age and contribute to both cancer development and aging. It is worth noting that mutations can also be present in normal states and other diseases [40,41]. Cancer risk factors can be broadly classified into three groups: intrinsic factors (such as DNA errors), and two categories of non-intrinsic factors (endogenous factors like immune responses, metabolic changes, inflammation, DNA repair problems, aging, and exogenous factors like viruses, chemicals, radiation, lifestyle, etc.) [42]. Non-mutagenic factors can also play a significant role in cancer development.

Allan Balmain reported that mutations are important but not sufficient for carcinogenesis [43]. He suggests that non-genotoxic factors that induce cancer have a more substantial impact than previously thought.

In their study [44], the authors demonstrated that certain metabolites, such as 2-hydroxyglutarate, can have oncogenic effects by altering cell signaling and disrupting cell differentiation.

There are two types of genes involved in cancer development, often referred to as “brake and gas pedals”. Proto-oncogenes regulate cell growth and proliferation, and when mutated, they become oncogenes (e.g., *Ras*). On the other hand, tumor suppressor genes (e.g., *p53*, *Rb*) act as guardians by detecting mutations and repairing damages or halting the cell cycle until cell death. Suppressor genes can also reverse mutagenic effects [45]. 

The year 2023 marked the 100th anniversary of Otto Warburg’s discovery that cancer cells preferentially generate energy from glucose through “aerobic glycolysis” in the cytosol rather than using mitochondria, resulting in lactate formation [46]. Warburg proposed that irreversible damage to respiration is the cause of cancer [47,48]. Thus, an alternative perspective suggests that the origin of cancer lies in metabolic alterations, particularly in energy metabolism with dysfunctional mitochondria [49,50]. Thomas N. Seyfried referred to cancer as a “metabolic disease” and suggested that mutations in *p53* and *Ras* impair mitochondrial respiratory function. Cells with compromised respiration are more prone to genome instability and transformation into malignancy. 

**Figure 2:**
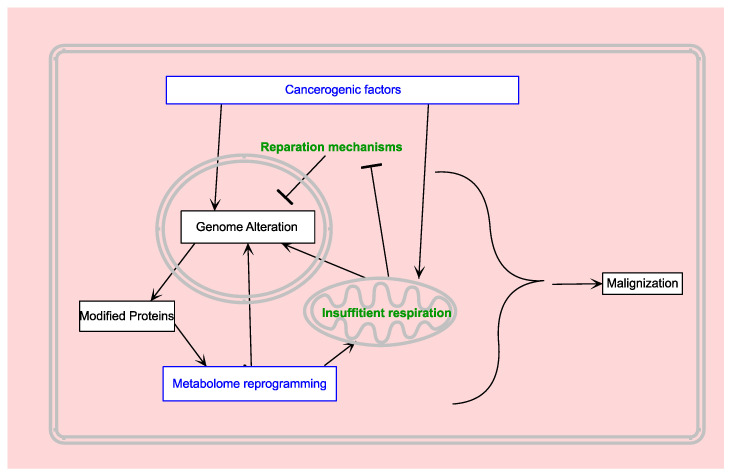
Basic routes to cancerogenesis.

Metabolically reprogrammed cells, especially those with impaired mitochondria and altered energy metabolism, become more susceptible to genetic mutations. Genetic mutations, in turn, drive profound metabolic reprogramming. This exemplifies a pathological feedback loop (see Figure 2).

Additionally, cancer cells have the ability to communicate with normal cells through signaling molecules, allowing them to reprogram the metabolism of surrounding cells to promote tumor growth [51]. Tumors produce a pro-tumoral and immunosuppressive microenvironment [52].

Lipidomics studies play a crucial role in understanding the pathogenesis of various diseases [53]. Alterations in lipidomics pathways can serve as biomarkers or therapeutic targets for the treatment of disorders [54]. Lipidomics and metabolomics research contribute to personalized medicine approaches [55].

Numerous studies have suggested that cancer cells undergo significant changes in lipid metabolism. Lipids serve as essential energy sources, and alterations in lipid metabolism align with the concept of disrupted energy metabolism in cancer cells. Understanding the unique lipidomic characteristics of cancer cells is important for elucidating their role in cancer development and progression.

Lipids are targeted compounds in ferroptosis; this is why the specific characteristic of lipidomics in cancer is important to explain.

Munir et al. discussed the metabolic adaptation of cancer cells in response to the harsh tumor microenvironment and the requirement for de novo fatty acid biosynthesis [56].

Koundouros and Poulogiannis showed the role of fatty acids biosynthesis in cancer metabolism [57].

Khan et al. explained lipid metabolism reprogramming in cancer progression [58].

Pakiet et al. [59] emphasized the potential of specific saturated and monounsaturated lipids, such as PC-16:0/16:1, as possible biomarkers in colorectal cancer.

Beloribi-Djefaflia et al. [60] proposed disrupting lipid metabolism in cancer cells, through targeting enzymes, receptors, or bioactive lipids, as a potential approach for cancer treatment.

Metabolic and lipidomic reprogramming plays a significant role in the development and progression of cancer throughout the entire organism. Therefore, an effective approach to treating cancer is to disrupt the pathological feedback loop by inducing reverse metabolic reprogramming, which includes altering the feeding of cancer cells or the overall nutrition of the organism.

It is essential to differentiate between reprogramming and reverse-reprogramming. Pathological reprogramming refers to the malignant transformation of normal cells, as well as alterations in the lipidome and metabolome during carcinogenesis. Therapeutic reprogramming, on the other hand, aims to decrease malignancy, increase sensitivity to cell death, and ultimately restore cells to a normal state.

Understanding metabolic and lipidomic reprogramming in cancer cells is crucial for identifying more effective strategies for inducing ferroptosis in cancer treatment. The first step is to study the metabolome and lipidome characteristics of cancer cells and identify the target genes and pathways that can be manipulated to induce ferroptosis as a therapeutic approach.

This review focuses on the two most selective pathways related to lipid metabolism in cancer cells and prospective targets for ferroptosis sensitization, namely de novo lipogenesis and ketolysis. 

The connections between de novo lipogenesis inhibition and ferroptosis sensitization are exemplified in the deep genetics and lipidomics study [61].

### 2.1. Genes Involved in Cancerogenesis That Regulate De Novo Lipogenesis in Cancer

In cancer cells, several genes involved in cancerogenesis regulate de novo lipogenesis, which plays a crucial role in lipid metabolism and can impact ferroptosis resistance and sensitivity. These genes are responsible for the production of saturated fatty acids (SFAs) and monounsaturated fatty acids (MUFAs), which are essential components of cell membranes.

De novo lipogenesis is selective for cancer cells because, among normal cells, only adipocytes and hepatocytes are primarily involved in this process [62]. 

**Figure 3:**
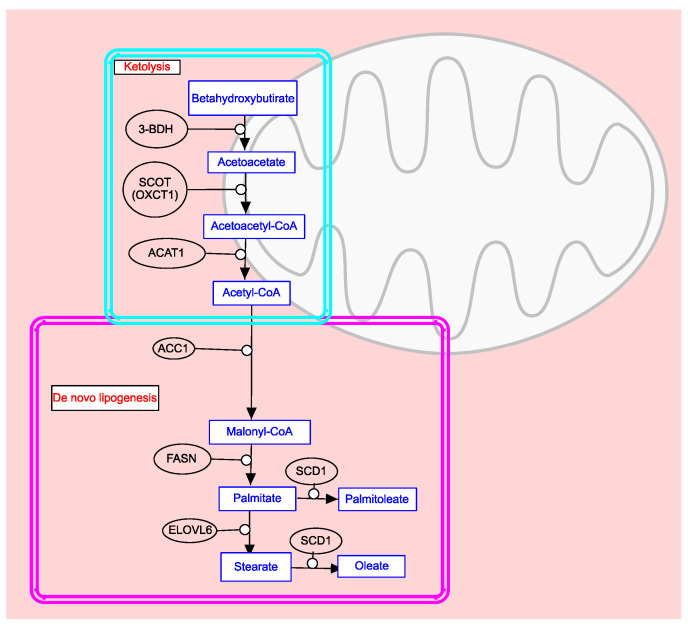
Ketolysis (BioCyc ID: PWY66-368 [64]) and de novo FA biosynthesis (based on Reactome fatty acid metabolism pathway Id: R-HSA-8978868.7 [65]).

Among the genes regulating de novo lipogenesis in cancer cells, ACC1 (acetyl CoA carboxylase) is a key enzyme involved in fatty acid synthesis [63]. It catalyzes the carboxylation of acetyl-CoA to malonyl-CoA (Figure 3).

FASN (fatty acid synthase) is another enzyme involved in de novo lipogenesis. It catalyzes the conversion of malonyl-CoA to palmitate acid in the cytoplasm of cells [66]. *FASN* is a proto-oncogene that participates in membrane biosynthesis, cell proliferation, cell invasion, metastasis, lipid raft construction, immune evasion, recruitment of M2 macrophages and T regulatory cells, resistance to programmed cell death, and alterations in cell energetics [67].

SCD1 (stearoyl-CoA desaturase-1) is located in the endoplasmic reticulum and contains iron in its structure. It catalyzes the formation of a double bond in the fatty acid chain between the ninth and tenth carbon (Figure 3) [68]. SCD1 plays a crucial role in cancer progression and contributes to tumor aggressiveness [69]. Its expression levels vary across different cancer types [70]. 

ELOVL6 (elongation of very long chain fatty acids protein 6) is situated in the microsomes of the endoplasmic reticulum and is responsible for elongating SFAs and MUFAs containing 12, 14, and 16 carbons (Figure 3) [71]. Overexpression of *ELOVL6* is associated with poor prognosis in various types of cancer, including liver cancer, breast cancer, and head and neck squamous cell carcinoma [72,73,74] (Table 1).

### 2.2. Ketolysis Is Critical for Acetyl-CoA Production in Cancer

In addition to de novo lipogenesis, there is another specific pathway in cancer cells, and it generates acetyl-CoA for lipid synthesis, known as ketolysis (Figure 3). Israël and Schwartz [75,76] suggest that the such production of acetyl-CoA is essential for cancer cells due to damaged mitochondria metabolism, in which oxidative phosphorylation is inhibited. Inhibition of the oxidative phosphorylation process is a phenomenon known as the Warburg effect. Furthermore, the production of acetyl-CoA through β-oxidation is suppressed in cancer cells by the presence of malonyl-CoA derived from de novo lipogenesis. Ketolysis occurs within the mitochondria and is considered the “Achilles heel” of cancer cells [75]. The key enzymes involved in ketolysis are beta hydroxybutyrate dehydrogenase (3-BDH), succinyl-CoA: 3-oxoacid-CoA transferase (SCOT or OXCT1), and acetyl-Coenzyme A acetyltransferase 1 (ACAT1) (Figure 3). Therefore, ketolysis serves as the primary precursor pathway for de novo lipogenesis in cancer cells.

## 3. Ferroptosis Related Genes and Proteins as Therapeutic Targets

There are many key genes associated with ferroptosis, including *GPX4*, *p53*, *ACSL4*, *LPCAT3*, *LOX*, *TRF1*, *Ferritin*, *Ferroportin*, *HO-1*, *HSPB1*, *NCOA4*, *FSP1*, *GCH1*, and *NRF2*, among others. Numerous studies have investigated the role of these ferroptosis-related genes in different types of cancer. There are some reviews related to this topic [77,78].

In this section, we will discuss several genes that hold importance as therapeutic targets (see Table 2).

*ACSL4* is involved in the conversion of long-chain polyunsaturated fatty acids (PUFAs), such as arachidonic acid and adrenic acid, to CoA-PUFAs [22,79]. 

*LPCAT3*, also known as Lysophospholipid Acyltransferase 5, regulates the incorporation of PUFAs into the cell membrane. It was identified through haploid genetics screening as being active in ferroptosis induced by RSL3 and ML162 [11].

*NOXs* (*NADPH oxidases*) were implicated in the pioneering study of ferroptosis, which reported that erastin-induced cell death was mediated by reactive oxygen species (ROS) produced by NOX enzymes [1]. The paper reported that ferroptosis caused by erastin was blocked in Calu-1 cells via inhibition of NOX enzymes. *NOX1* is expressed more than *NOX4* in this type of cell. 

Lambeth et al. suggested that NOX enzymes produce ROS in a highly controlled manner [80]. 

Additionally, proteins like Rac1 and Rac2 are involved in the activation of NOX enzymes.

*Lipoxygenases* (*LOXs*) play a significant role in lipid peroxidation during ferroptosis. LOXs are a class of iron-containing enzymes that catalyze the addition of two oxygen atoms to polyunsaturated fatty acids in a stereospecific manner. Yang et al. reported that *LOXs* coordinate ferroptosis induced by erastin by catalyzing the formation of fatty acid hydroperoxides, which are further transformed into eicosanoids such as leukotrienes. The involvement of *LOXs* in the peroxidation of PUFAs, leading to ferroptotic cell death, occurs when cellular antioxidant resources, such as reduced glutathione (GSH), are depleted [81]. 

Inhibition of *12/15-LOX* or siRNA-mediated suppression of *LOXs* prevents ferroptosis, while overexpression of *ALOX15* sensitizes cells to ferroptosis inducers [82]. *ALOX15* regulates the peroxidation of arachidonic acid (ω-6), eicosapentaenoic acid (ω-3), and docosahexaenoic acid (ω-3) [83].

Overexpression of *15-LOX-1* (*ALOX15*) reduced the LD50 from 6.8 μM to 0.5 μM in RSL3-induced ferroptosis and from 6.6 μM to 1.7 μM in erastin-induced ferroptosis [84]. 

Furthermore, 15LOX was found to peroxidize PUFA-phospholipids exclusively in the PEBP1/15LOX complex [24].

*P450 oxidoreductase* (*POR*) was identified as a proferroptotic factor in a CRISPR study. Exhaustion of POR suppressed ferroptosis induced by erastin, FIN56, FINO2, and BSO [85]. POR provides electrons for the catalytic peroxidation of PUFA-phospholipids. The electron acceptor in this reaction requires further investigation. 

*MS4A15* decreases luminal Ca^2+^ levels and inhibits ferroptosis by altering the lipid composition, enriching monounsaturated fatty acid-phospholipids (MUFA-PLs) and MUFA plasmalogen ether lipids while restricting polyunsaturated fatty acid (PUFA)-lipids [86]. Depletion of Ca^2+^ blocks lipid elongation and preserves the saturation of ether lipids, making phospholipids more resistant to lipid peroxidation and, consequently, to ferroptosis.

Zou et al. described lipid differences in cancer cell lines that are sensitive to ferroptosis [87]. They identified the key role of *HIF-2α* in this sensitivity. In renal clear-cell carcinomas (CCCs), *HIF-2α* selectively enhances PUFA-phospholipids, which are sensitive to reactive oxygen species (ROS), by overexpressing hypoxia-inducible lipid droplet-associated protein (HILPDA).

The enzyme HO-1 has a contradictory role in cancer. Initially, it increases cancer progression by defending cells against ROS, but high levels of ROS influence HO-1 to produce more iron (II) through heme destruction, leading to lipid peroxidation [88].

Knockdown of the CD71 (Transferrin receptor 1) gene, which encodes the transferrin receptor, has been shown to increase resistance to ferroptosis induced by erastin [6]. The 3F3-FMA antibody, targeting the Trf1 antigen, has been used as a marker of sensitivity to ferroptosis [89]. Trf1 is responsible for supplying iron for cellular metabolism. While cancer cells require iron for their functions, the antibody targeting Trf1 has demonstrated anti-cancer effects [90]. However, it is important to note that iron is also necessary for the process of ferroptosis. In comparison to normal cells, cancer cells, particularly cancer stem cells, exhibit a higher dependence on iron [91]. Thus, Trf1 is a possible vulnerability of cancer cells.

Genetic inactivation of *SCD1* in A549 cells has been shown to increase their sensitivity to RSL3, while overexpression of *SCD1* in H358 cells has been shown to suppress RSL3-induced ferroptosis [92]. Inhibition of *SCD1* in ovarian cancer stem cells leads to ferroptosis [93,94].

*HMG-CoA reductase* (*HMGCR*) plays an important role in the synthesis of GPX4 [20] and CoQ10 [16], both of which have a preventive effect on ferroptosis. Additionally, HMGCR catalyzes the biosynthesis of cholesterol. Incorporating cholesterol into cell membranes can serve as a biophysical barrier to the propagation of lipid ROS.

**Table 2:** Genes involved in ferroptosis.

Genes Regulate Ferroptosis	Influence	Metabolic and Lipidomic Changes	Reference
*GPX4*	Suppressor	Recovery of peroxidized lipids	[10,20,95]
*ACSL4*	Sensitizer	Activation of the long chain PUFAs by CoA before incorporation into the lipid membrane structure	[22]
*LPCAT3*	Sensitizer	Incorporation of the long chain PUFAs into the membrane phospholipids	[11]
*ALOX15*	Sensitizer	Peroxidation of the AA and AdA	[84]
*NOX*	Promoter	Producing ROS and increasing of the membrane PUFA-PLs peroxidation	[1]
*P450*	Sensitizer	Increasing the membrane PUFA-PLs peroxidation	[85]
*MS4A15*	Suppressor	Elevation MUFA-PL and plasmalogen ether PL, limiting PUFA-PL	[86]
*HIF-2α*	Sensitizer	Enhancement of PUFA-PL	[87]
*HO-1*	Sensitizer	Elevation of lipid peroxidation	[88]
*CD71(TfR1)*	Sensitizer	Elevation of LIP	[89]
*FASN*	Suppressor	Increasing SFAs and MUFAs in lipids, limiting PUFA-PL	[61]
*SCD1*	Suppressor	Increasing MUFA/SFA ratio in lipids, limiting PUFA-PL	[70,94]
*HMGCR*	Suppressor	Elevation of the level of CoQ10 (antioxidant) and production of IPP, which participate in the building of GPX4	[16,20,30]

## 4. Lipidomic Changes at Ferroptosis: Keys to Increase Sensitivity to Ferroptosis

Membrane phosphatidylethanolamines (PEs) were initially discovered as the primary target of lipid peroxidation in RSL3-induced ferroptosis. Kagan et al. demonstrated that arachidonate and adrenate phosphatidylethanolamines (PE-(C18:0/C20:4), PE-(C18:0/C22:4)) undergo peroxidation, resulting in the following peroxidation products:

PE-(C18:0/C20:4+2[O])
PE-(C18:0/C20:4+3[O])
PE-(C18:0/C22:4+2[O])
PE-(C18:0/C22:4+3[O]) [27].

The study found that the highest levels of oxidized phosphatidylethanolamines (oxyPE) and oxidized phosphatidylserines (oxyPS) were observed in ferroptosis compared to apoptosis, necroptosis, non-canonical pyroptosis, and canonical pyroptosis. OxyPI was more abundant in ferroptosis than apoptosis, but was not detected in other forms of cell death. OxyPC, on the other hand, was present in all types of cell death [96].

In HT1080 cells, erastin-induced ferroptosis was associated with an increase in lysoPC levels [10]. The lipidomics profile in erastin-induced ferroptosis differs from that of RSL3-induced ferroptosis. It can be hypothesized that erastin partially activates phospholipase A2.

Numerous studies have suggested that differences in lipid composition play a crucial role in susceptibility to ferroptosis, particularly the involvement of polyunsaturated fatty acids (PUFAs). Lin et al. [97] analyzed the key lipid metabolism features associated with ferroptosis sensitivity.

Peroxisomes and polyunsaturated ether phospholipids (PUFA-ePLs) have been found to significantly influence ferroptosis sensitivity. The reduction of PUFA-ePLs levels promotes a resistant state against ferroptosis and regression of cancer therapy in clear-cell renal-cell carcinoma (ccRCC) [98].

Magtanong et al. observed that monounsaturated fatty acids (MUFAs) confer resistance to ferroptosis by incorporating them into membrane phospholipids (PLs) through the action of ACSL3. In addition, MUFAs help to collect SFAs [99]. Ether-MUFAs have correspondingly been shown to inhibit ferroptosis by Xin and Schick (2022) [86], whereas ether-PUFAs promote ferroptosis.

SFAs and SFA-PLs lack double bonds and, therefore, cannot participate in lipid peroxidation, rendering them resistant to ferroptosis. They act as mechanical (biophysical) barriers against the propagation of lipid reactive oxygen species (ROS).

**Figure 4:**
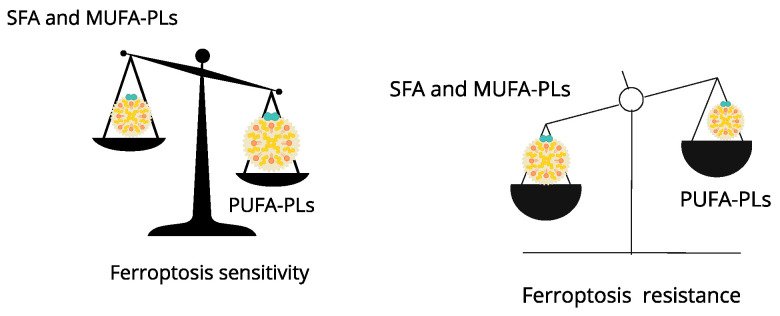
Balance of membrane phospholipids modulate susceptibility to ferroptosis.

On the other hand, the presence of PUFAs in membrane PLs has been shown to increase susceptibility to ferroptosis, while MUFAs and SFAs correspond to a resistant state [100,101] (Figure 4). 

**Figure 5:**
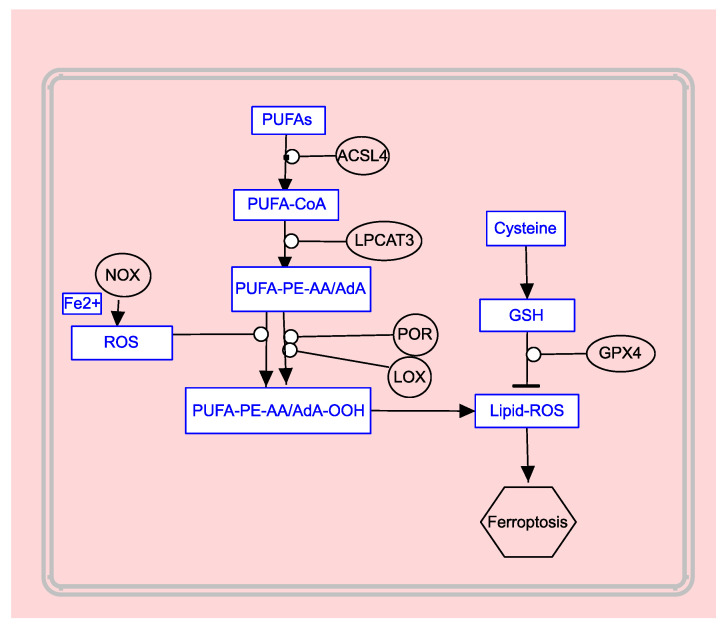
PUFAs drive ferroptosis (based on the KEGG hsa04216 Ferroptosis).

The scheme showing PUFAs participation in ferroptosis is presented in Figure 5.

## 5. Chemical Modulators of Ferroptosis

### 5.1. Ferroptosis Inducers

Yang et al. concluded that ferroptosis inducers can be classified into two categories. The first class inhibits GPX4 through GSH depletion, as in the case of erastin. The second class inhibits GPX4 by binding directly to it, such as in RSL3 [10].

Erastin was discovered to selectively induce non-apoptotic cell death in cells expressing the small T oncoprotein and oncogenic Ras [4].

Similar to glutamate, erastin blocks the transport of cysteine into the cell by inhibiting the cystine/glutamate antiporter (System xc^-^). This leads to depletion of antioxidants, resulting in oxidative, iron-dependent cell death [1].

Erastin significantly depletes both reduced glutathione (GSH) and oxidized glutathione (GSSG) [10].

RSL3, on the other hand, does not affect GSH levels but covalently binds to and inhibits GPX4 directly [10].

BSO is another compound that reduces GSH levels [10].

Lippmann et al. proposed a new treatment approach for hepatocellular carcinoma, involving the combination of auranofin and BSO or erastin and BSO to induce ferroptosis. This approach utilizes both canonical (GPX4 suppression) and non-canonical pathways (expression of Nrf2 and enhancement of *HO-1*) [102].

Sulfasalazine and sorafenib are clinically proven drugs that have been approved by the FDA. These drugs inhibit system xc- and induce ferroptosis [103]. 

Sulfasalazine was originally used as an anti-inflammatory agent, while sorafenib is an anticancer drug. Since these drugs are already approved for medical use, they have the potential to be immediately tested and utilized for ferroptosis-based cancer treatment, following the new strategies proposed in the current review with sensitizers. This provides an accelerated way to clinical implementation.

Several natural compounds have been associated with the induction of ferroptosis [104]. The mechanism of artemisinin helps produce reactive oxygen species (ROS) through iron metabolism. Artesunate increases the labile iron pool through ferritinophagy in lysosomes. Dihydroartemisinin leads to a decrease in reduced glutathione (GSH) levels and an increase in lipid ROS. Gallic acid inhibits GPX4. Erianin and salinomycin elevate the intracellular iron pool.

A chemoinformatic analysis of different ferroptosis modulators is presented in the review [105]. Natural phenolic compounds, such as typhaneoside, robustaflavone A, amentoflavone, and erianin (at μM levels), have demonstrated antitumor properties by inducing ferroptosis in vitro and in vivo. However, some compounds like apigenin, baicalin, resveratrol, and curcumin may have a dual effect depending on the cell type or composition of the formulation.

N6F11 is a novel selective ferroptosis inducer that does not suppress immune cells. It activates TRIM25, leading to the degradation of GPX4 [30,31,32].

The active compounds that induce ferroptosis are listed in Table 3.

### 5.2. Ferroptosis Inhibitors

Ferrostatin-1 has been found to inhibit lipid peroxidation without decreasing mitochondrial reactive oxygen species or lysosomal membrane permeability [8].

It functions as a scavenger of free radicals [107]. The proposed mechanism of ferrostatin-1 is primarily as a radical-trapping antioxidant rather than as an inhibitor of lipoxygenases [108]. However, there is evidence suggesting that it blocks HpETE-PE formation by the 15LOX/PEBP1 enzymatic couple [109]. This suggests that the ultimate executioners of ferroptosis have not yet been identified.

Several natural compounds exhibit anti-ferroptotic effects through ROS scavenging, iron chelation, or the expression of GPX4 and NRF2 [105]. Many polyphenols, such as galangin, kaempferol, naringenin, quercetin, green tea catechins, and fisetin, act as ferroptosis defenders at micromolar concentrations, thereby exerting a neuroprotective effect.

PUFA-PLs are the main participant in peroxidation in ferroptosis; however, omega-3 PUFAs support transcription factors which enhance antioxidant systems and reduce lipid peroxidation via ferroptosis [110]. Therefore, they have potential as ferroptosis inhibitors.

NADPH and niacin (Vitamin B3) are relevant in this context. Niacin, including nicotinic acid and nicotinamide, serves as a precursor of NADPH. Niacin deficiency activates NADPH oxidase and increases ROS in HaCaT keratinocytes [111]. 

Therefore, it is logical to test niacin (nicotinic acid, nicotinamide, and NADPH) as a potential anti-ferroptotic agent and a prospective mitigator of side effects associated with ferroptotic anticancer therapy.

## 6. Therapeutic Strategies Based on the Metabolome and Lipidome Reprogramming

Ferroptosis is a promising alternative for the treatment of cancers that are resistant to chemotherapy [112].

Several cell lines have been found to be susceptible to ferroptosis, including A-673 (human muscle sarcoma) cells, SK-BR-3 (human breast cancer) cells, Huh-7 (hepatocyte-derived carcinoma) cells, and SK-LMS-1 (human leiomyosarcoma) cells [89]. Overexpression of Trf1 serves as a marker of sensitivity to ferroptosis, as it leads to increased iron uptake by cells. ACSL4 is overexpressed in HepG2 and HL60 cell lines, which are sensitive to ferroptosis [17]. On the other hand, ferroptosis-resistant cell lines such as LNCaP and K562 exhibit low expression of ACSL4. Increased de novo lipogenesis has been associated with greater resistance to lipid peroxidation [113], with cells like LNCaP and HCT116 cells showing the highest resistance.

### 6.1. De Novo Lipogenesis Pathway as Targets for Sensitization to Ferroptosis

Increased de novo lipid biosynthesis is considered one of the important characteristics of cancer cells [62,114].

Normally, all lipids (saturated and unsaturated) in mammalians are present in the body due to food intake.

**Figure 6:**
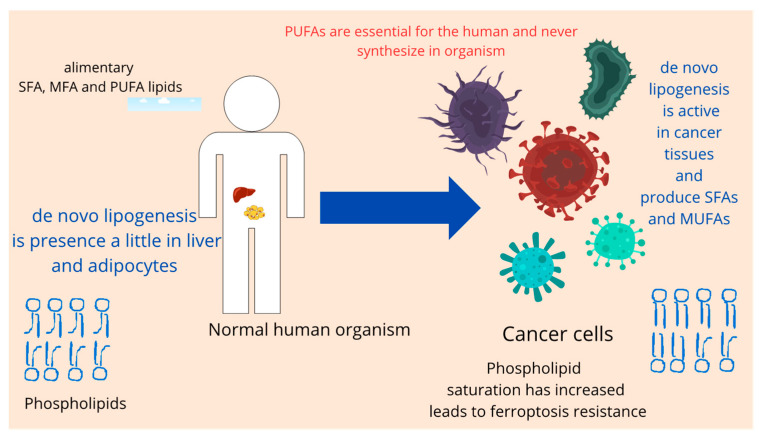
Increasing DNL is a cancer hallmark.

Saturated fatty acids (SFAs) and monounsaturated fatty acids (MUFAs) can be synthesized through de novo lipogenesis (DNL) from carbohydrates, which is a metabolic feature observed in many cancer cells. Under normal conditions, DNL is balanced between the liver and adipose tissue. However, in pathologies such as obesity and insulin resistance, this balance is disrupted, and DNL is primarily shifted towards the liver [115]. De novo lipogenesis is also observed in various other tissues in malignancy [62] (Figure 6). Therefore, enzymes involved in de novo lipogenesis represent selective targets against cancer cells because they are typically active in only a few normal cell types.

PUFAs (polyunsaturated fatty acids) are essential components of food and cannot typically be synthesized in mammals [116].

In vivo and in vitro studies have shown that reducing de novo lipogenesis leads to increased assimilation of nutritious PUFAs [117]. Interruption of de novo lipogenesis can impact the exploitation of PUFAs from the diet. 

Tumors with high aggressiveness often exhibit high rates of de novo lipid biosynthesis. Rysman et al., based on mass spectrometry analysis, demonstrated that clinical tumor tissues with high lipogenesis have increased lipid saturation compared to non-lipogenic tumors. This increased saturation makes them more resistant to lipid peroxidation. These changes in membrane lipid saturation were observed in prostate cancer cell lines (LNCaP, 2Rv1, PC-3, and Du145), breast cancer (BT474), and colorectal cancer (HCT116). The most significant elevation in saturation was observed in LNCaP and HCT116 cells [113]. 

Ferroptosis-resistant cell lines pose a significant challenge. This resistance can be attributed to the increased saturation of cell membrane lipids, as observed in LNCaP and HCT116 cells.

One paper [113] described how the lipogenesis inhibitor soraphen A, or targeting lipogenic enzymes with siRNA, leads to lower levels of saturated and monounsaturated phospholipids and an increase in polyunsaturated lipids. Soraphen A specifically inhibits ACC1, and authors [118] have proposed ACC1 as a potential biomarker and target in non-small-cell lung cancer (NSCLC). New ACC1 inhibitors, such as ND-646 and its derivatives, have been discovered and studied in A549 cells, showing inhibition of cancer growth with an IC50 of 9–17 nM.

Other known ACC inhibitors include CP-640186, haloxyfop, sethoxydim, and moiramide B [63].

Inhibition of FASN (fatty acid synthase) reduces (SFAs) and (MUFAs), regulating the process of including PUFAs into phospholipids (PLs) of mutant KRAS lung cancer cells, and increasing their sensitivity to ferroptosis [89].

Overexpression of FASN elevates SFAs and MUFAs lipid levels and reduces PUFA lipids in cancer cells, in opposition to normal cells [113]. It supports cancer cell resistance to ROS. Inhibition of FASN can have a significant impact.

FASN inhibitors selectively induce programmed cell death in different tumor cells while sparing normal cells since only a small number of normal cells depend on FASN [119]. GSK2194069 was proposed as a FASN inhibitor (IC50 7.7 nM).

Several FASN inhibitors have been discovered [120], including C75, orlistat, the polyphenol epigallocatechin-3-gallate from green tea, and flavonoids such as luteolin, quercetin, and kaempferol. C75 is a synthetic analog of orlistat. FASN inhibitors are of interest in studying the reprogramming of the lipidome to enhance ferroptosis susceptibility, particularly in combination with ferroptosis inducers.

FASN inhibitors are of interest in the reprogramming of the lipidome to elucidate ferroptosis susceptibility, for further combination with ferroptosis inducers.

Authors [94] have proposed a complex therapy strategy involving the simultaneous application of SCD1 suppressors and ferroptosis inducers.

New inhibitors of SCD1 (SSI-1, SSI-2, SSI-3, and SSI-4) have been discovered using advanced chemoinformatic drug development [121]. 

Pharmacological inactivation of SCD1 with CVT11127 in STK11/KEAP1 double knockout lung adenocarcinoma cells has been shown to reverse resistance and make these cells sensitive to ferroptosis induced by erastin [92]. SSI-4 and icomidocholic acid (aramchol) are inhibitors of SCD1 which have successfully finished clinical trials.

SSI-4 and icomidocholic acid (aramchol) are inhibitors of SCD1 that have successfully completed clinical trials. Inhibition of SCD1 leads to a decrease in the ratio of MUFAs to SFAs, an increase in PUFAs, and a promotion of a less aggressive survival phenotype. SCD1 upregulates the MUFAs/SFAs ratio in lipids, while PUFAs are significantly downregulated [122]. Inhibition of SCD1 can lead to different types of programmed cell death [70,123].

Several thorough reviews have also focused on the inhibition of de novo fatty acid biosynthesis as an anti-cancer therapy [57,67,124,125].

### 6.2. Ketolysis Pathway as a Target for Ferroptosis Sensitisation

It was noted earlier that the primary substrate for de novo lipogenesis in cancer cells, which is ultimately produced by ketolysis, is acetyl-CoA [75,76]. There are three more sources of acetyl-CoA. The first is derived from glucose through pyruvate. Pyruvate forms acetyl-CoA (via catalysis by the pyruvate dehydrogenase complex (PDH)). In tumor cells, it is deactivated. The second source is from β-oxidation of FA (inhibited in cancer). The final source is from exogenous acetate (catalyzed by acetyl-CoA synthase (ACS)). Authors [126] have proposed to inactivate the latter pathway using allicine or orotate. In contrast to normal cells, in cancer cells, all three of these sources are unavailable, and only ketolysis is a viable pathway for acetyl-CoA production. Pharmacological intervention could possibly ultimately shut this door. Depletion of acetyl-CoA disrupts many metabolic pathways, including fatty acid and cholesterol metabolism; thus, normal cells will have better opportunity for survival.

Genes that support the production of acetyl-CoA through ketolysis are important targets for cancer treatment. One of the main enzymes involved in ketolysis is SCOT (succinyl-CoA: 3-oxoacid-CoA transferase). Abolhassani et al. suggested that inhibiting SCOT and ketolysis can suppress tumor growth [127]. The authors studied several compounds, including lithostat, 2-epigallocatechin, 3-alpha R lipoic acid, hydroxycitrate (from Garcinia Cambogia), and allicin, and demonstrated tumor inhibition comparable to 70% cisplatin treatment.

M. Israel et al. provided an in-depth explanation of the role of ketolysis in cancer progression and the anticancer mechanisms of ketolysis inhibitors [126].

If ketolysis is inhibited, acetyl-CoA will be depleted, so antiferroptotic de novo lipogenesis will be blocked, and production of SFAs and MUFAs will be depleted. Furthermore, its level in membrane PL will be reduced. The antiferroptotic mevalonate pathway will also be blocked, isopentyl pyrophosphate (IPP) will be depleted, GPX4 will crash, and cholesterol will also be decreased.

For incorporation of exogenous proferroptotic PUFAs into cell membrane PL, there is no need for acetyl-CoA. ACSL4 (acyl-CoA synthetase long chain family member 4) uses only CoA-SH, which is synthesized from pantothenate, for activation of PUFAS before integration into membrane PLs; for PUFAs, the door is open. 

Therefore, reprogramming tumor cells by inhibition of ketolysis will make more sensitive to ferroptosis state is key.

Dependence of ferroptosis sensitivity on ketolysis inhibition has not been shown in the literature as yet.

One can consider ketolysis inhibitors as ferroptosis sensitizers. Thus, the combination of inhibitors of ketolysis and ferroptosis inducers promises to have a synergetic effect.

Compounds-inhibitors of de novo lipogenesis and ketolysis are presented in Table 4.

### 6.3. Ferroptosis Sensitizers Which Target Genes Involved to PUFA-PLs Biosynthesis and Metabolism

The PPARδ (peroxisome proliferator-activated receptor delta) activator, L-165041, has been shown to increase the expression of ACSL4 in vivo, specifically in hamster liver, as well as in primary human, hamster, and mouse hepatocytes [128]. Activation of ACSL4 can lead to the formation of a ferroptosis-sensitive lipidome.

Activation of LXR (liver X receptor) cause overexpression of LPCAT3 [129]. Therefore, LXR activators, such as saikosaponin A [130] and 24-hydroxycholesterol [131], are prospects for sensitization to ferroptosis.

ML329, an inhibitor of breast cancer stem cells, has been discussed as an activator of FADS2, which can increase the levels of PUFA-PLs (phospholipids containing polyunsaturated fatty acids) in cancer cells, thereby increasing their unsaturation [25]. This could potentially increase the sensitivity of cells to ferroptosis.

Darapladib, an Lp-PLA2 inhibitor, can protect PUFA-PLs from hydrolysis using phospholipase, making cells more susceptible to ferroptosis by elevating the levels of phosphatidylethanolamine at the expense of lysophosphatidylethanolamines [132]. Genetic suppression of PLA2G7, which encodes Lp-PLA2, has shown similar results.

Etomoxir is an irreversible inhibitor of carnitine palmitoyltransferase 1a (CPT1a), a suppressor of PUFA β-oxidation [133]. Etomoxir has been shown to enhance the ability of RSL3 to induce ferroptosis [27]. 

One possible explanation for the sensitization to ferroptosis caused by etomoxir is its inhibition of β-oxidation, which blocks the production of acetyl-CoA, a precursor of de novo lipogenesis.

Malonyl-CoA has been described as an inhibitor of CPT1 [134,135].

Therefore, a further “hack” would be to employ malonyl-CoA as a non-toxic ferroptosis sensitizer that reversibly inhibits CPT1.

### 6.4. Ferroptosis Sensitizers Acting on Different Lipid Metabolism Pathways Reprogramming 

Hemin, an iron-containing compound, has been shown to boost ferroptosis in platelets [136]. In lung cancer cells, hemin induces ferroptosis by increasing the effectiveness of reactive oxygen species (ROS) in lipid peroxidation [137]. The induction of ferroptosis by erastin, another ferroptosis inducer, is enhanced by hemin and CO-releasing molecules (CORM) [13].

Hemoglobin, under oxidative stress, releases the heme prosthetic group, which has been shown to be cytotoxic due to its ability to catalyze ROS formation. Heme can activate ferroptosis in human platelets [138].

Cetuximab, a monoclonal antibody targeting the epidermal growth factor receptor, suppresses the protective properties of the Nrf2/HO-1 pathway in KRAS mutant colorectal cancer. As a result, it supports RSL3-induced ferroptotic cell death [139].

A study demonstrated a synergistic effect between multiple inhibitors of HMGCR (hydroxymethylglutaryl-coenzyme A reductase), such as statins and RSL3 [25].

The histone deacetylase inhibitor (HDACi) romidepsin increases the sensitivity of SW13 cancer cells to erastin-induced ferroptosis by activating epithelial-mesenchymal transition (EMT) and downregulating ferroportin, thereby increasing iron levels and ROS production [140].

Table 5 is devoted to the compounds which are prospects for rendering cells ferroptosis-sensitive.

### 6.5. Redox Balance Reprogramming for Ferroptosis Sensitization

Reprogramming the redox equilibrium is a promising approach for inducing or preventing ferroptosis. Cells have enzymatic and non-enzymatic mechanisms for the peroxidation of free fatty acids (FAs) and polyunsaturated fatty acid-containing phospholipids (PUFA-PLs), but this process is balanced by antioxidant systems such as glutathione (GSH), CoQ10, and tetrhydrobiopterin (BH4).

#### 6.5.1. Activation of Enzymes Involved in Peroxidation

NOX (NADPH oxidase) activation.

Arachidonic acid (AA) can initiate NOX [141].

Phospholipids, particularly phosphatidic acids (PA), can activate NOX through protein kinase in cell-free experiments [142]. Dicapryl-sn-glycerol-3-phosphate (10:0) works at a concentration of 10 µM, and PA in combination with diacylglycerol has a synergistic effect.

Arachidonic acid and the phosphorylation of p47phox by protein kinase C work together to activate NOX at a concentration of AA ranging from 1 to 5 µM under cell-free conditions [141]. Activation with a single AA was observed at higher concentrations of 50–100 µM. The anionic amphiphile SDS (sodium dodecyl sulfate), at levels ranging from 50 to 150 µM, can also initiate NOX activity in vitro. Additionally, 8,11,14-eicosatrienoic acid has a slight activation effect.

NADPH is depleted during CD38 activation [143]. The analog of calcitriol, 1alpha,25-dihydroxyvitamin D3, stimulates CD38 expression [144]. Inecalcitol, an analog of calcitriol, can also induce CD38 overexpression.

It is possible to test 1alpha,25-dihydroxyvitamin D3 (calcitriol), or its analog inecalcitol, as ferroptosis sensitizers. They reduce NADPH levels, thereby increasing NOX activity and sensitivity to ferroptosis. Calcitriol is used as an anticancer agent, but can cause hypercalcemia as a side effect. Inecalcitol has a lower incidence of side effects. Combining them with ferroptosis inducers may reduce the effective dose (ED50) required, and thus the corresponding toxic effects.

MnTE-2-PyP has been combined with radiation for the treatment of prostate cancer. It suppresses enzymes in the pentose phosphate pathway, depleting NADPH and decreasing GSH/GSSG ratios [145]. Depletion of glutathione is evidence of increased ferroptosis sensitivity. MnTE-2-PyP can be tested as a ferroptosis sensitizer.

LOX (lipoxygenase) activation:

(E)-1-(7-benzylidene-3-phenyl-3,3a,4,5,6,7-hexahydroindazol-2-yl)-2-(4-methylpiperazin-1-yl)ethanone (PKUMDL_MH_1001) has been proposed as an activator of ALOX15. The authors claim that ferroptosis is related to the cell membrane-localized enzyme. They have shown that the LOX activator sensitizes cells to ferroptosis at low doses of erastin and RSL3 [80].

An additional study found a synergistic stimulation of ferroptosis with locostatin [24]. Locostatin releases (activates) PEBP1 for interaction with 15LOX.

Table 6 presents genes that regulate lipid peroxidation and corresponding agonists.

#### 6.5.2. “Relaxing” Oxidative Stress: Lipidome Reprogramming as New Alternative Paradoxal Key to Ferroptosis Sensitization

Supplementation of polyunsaturated fatty acids (PUFAs) prior to ferroptosis-inducing treatment has been shown to sensitize cells. Omega-6 PUFAs are more prone to peroxidation than omega-3 PUFAs, making them more likely to contribute to ferroptosis and enhance its effects [110].

Ascorbic acid (vitamin C) has been used for sensitization to erastin-induced ferroptosis [147].

The authors described that the application of a gradual increase in the concentration of RSL3 led to the reprogramming of BT474 cells into an RSL3-resistant state [148]. This suggests that antioxidant treatment may have the potential to make cells more sensitive to ferroptosis.

Ferrostatin-1 and new generations of ferrostatins (such as SRS16-86 and SRS11-92) as well as natural antioxidants like eugenol, sulforaphane, and erucin are potential reprogrammers of tumor cells towards a ferroptosis-sensitive phenotype.

Thiothriazoline [149], thiometrizole (discovered at Zaporizhzia State Medical and Pharmaceutical University in Ukraine), and other thio-1,2,4-triazoles [150,151,152,153,154,155] have been identified as highly effective antioxidants and need to be tested as potential agents for relaxing oxidative stress and reprogramming the lipidome towards elevated PUFA levels.

Thiothriazoline and thiometrizole act as antioxidants and scavengers of reactive oxygen species (ROS). Thiothriazoline has shown properties that seem to oppose the Warburg effect. The substance activates lactate dehydrogenase (LDH) and promotes the conversion of lactate to pyruvate, thereby reducing lactate acidosis and stimulating the Krebs cycle. Other thio-1,2,4-triazoles should be tested in a similar manner. Investigation into the influence of such opposing Warburg effects on cancer cells, particularly in terms of lipidome and phenotype, will be promising in cancer study and treatment.

Yousefian [156] et al. suggested that polyphenols such as berberine, thymoquinone, catechin, celastrol, apocynin, resveratrol, curcumin, hesperidin, G-hesperidin, and quercetin possess antioxidant properties as free radical scavengers and can inhibit the activity of NADPH oxidases.

### 6.6. General Strategies to “Hack” the Lipidome for Cancer Treatment by Ferroptosis 

Each cancer type has both common and specific characteristics in terms of resistances and vulnerabilities of the cancer cell lipidome and metabolome to ferroptosis.

Monitoring the elevation of PUFA levels during ferroptosis sensitization treatment can serve as a sign that the cells are ready for switching to ferroptosis-inducers therapy.

Reprogramming cancer cells to increase the levels of PUFAs in cell membrane phospholipids while decreasing saturated fatty acids (SFAs) and monounsaturated fatty acids (MUFAs) is necessary to enhance sensitivity to ferroptosis.

**Figure 7:**
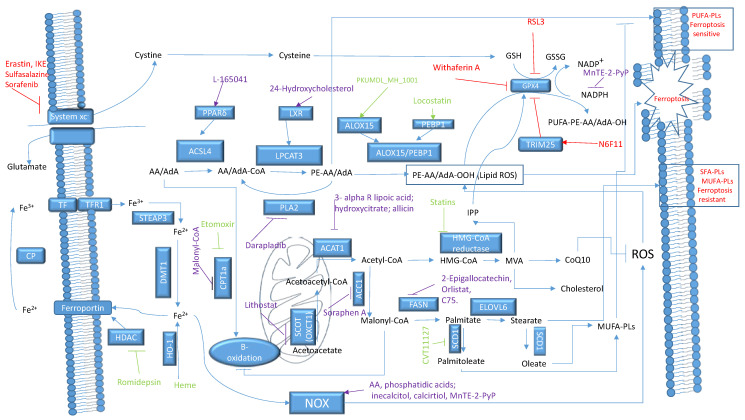
Ferroptosis sensitization pathways (developed based on KEGG hsa04216 Ferroptosis [157]). Red represents ferroptosis inducers. Green represents tested ferroptosis sensitizers. Violet represents potential ferroptosis sensitizers.

Figure 7 presents the most important tested and potential targets for ferroptosis sensitization. 

The following strategies can be employed:

- Assess all enzymes involved in lipid metabolism which promote the incorporation of MUFAs and SFAs into cell membrane phospholipids and inhibit these enzymes.
- Assess all enzymes involved in lipid metabolism which promote the incorporation of more PUFAs into cell membrane phospholipids and activate these enzymes.
- Block de novo lipogenesis by inhibiting the responsible enzymes.
- Block the production of Acetyl-CoA, which is the primary substrate for de novo lipogenesis, by inhibiting ketolysis.
- Activate enzymes that generate lipid ROS (such as NOX, LOX, COX, and POR) for synergistic use with ferroptosis inducers.
- Increase PUFA assimilation with nutrients.
- Preserve PUFAs (“relax” ROS) and accumulate them using antioxidants (ferrostatin, sulforaphane, erucin, etc.) or through reversible inhibition of NOX, LOX, COX, POR (for example, using niacin to decrease NOX activity).

The algorithm for scouting ferroptosis sensitizers for new drug development and therapeutic strategies involves the following steps:

- Screen substances that correspond to target genes.
- Assess changes in lipidomics after cell culture treatment (changes in PUFA-PL/MUFA-PL/SFA-PL).
- Determine the order of treatment (pre- or co-treatment).
- Check cell viability after ferroptosis induction.
- Conduct toxicology studies and use xenograft models.
- Proceed with clinical development.

Combinations of active pharmaceutical ingredients, whether used simultaneously or sequentially, can have more potent and targeted effects. Simultaneous combination therapy can lead to synergistic effects and help lower therapeutic concentrations. Sequential combination therapy may be used when one component reprograms the lipidome and metabolome to a more sensitive state, and another component induces ferroptosis. This approach can be an effective method to overcome drug resistance. Different classes of ferroptosis inducers can be combined, and sensitizers can be used together with inducers.

Given the need for personalized medical care, it is useful to conduct individual studies of lipid composition for each specific patient and their disease in order to select the optimal ferroptosis sensitizers.

## 7. Conclusions and Future Perspectives

Cancer cells have several vulnerabilities that make them more susceptible to ferroptosis-inducing therapy as compared to normal cells. These vulnerabilities can be targeted to selectively induce ferroptosis in cancer cells.

One specific vulnerability of cancer cells is their high reliance on de novo lipogenesis, a metabolic pathway involved in the synthesis of fatty acids. De novo lipogenesis is highly active in cancer cells, but almost entirely inactive in normal cells, except for liver cells and adipocytes [33,115]. Inhibiting de novo lipogenesis selectively targets cancer cells. 

In addition, cancer cells have limited alternative sources of acetyl-CoA, a primary metabolite in de novo lipogenesis.

Inhibition of acetyl-CoA production through the inactivation of ketolysis, which is responsible for generating acetyl-CoA, is specific to cancer cells. This is because other sources of acetyl-CoA, such as the Krebs cycle and fatty acid β-oxidation, are inhibited in cancer cells. Normal cells are safe during the inhibition of ketolysis because they have alternative open sources of acetyl-CoA [75,76,126,127].

These pathways generate antiferroptotic saturated fatty acids (SFAs) and monounsaturated fatty acids (MUFAs) that contribute to the composition of membrane phospholipids, making the membranes more resistant to ferroptosis. Acetyl-CoA is a precursor of the antiferroptotic mevalonate pathway, which builds CoQ10, GPX4, and cholesterol. All of this data serves as evidence that ketolysis is also an antiferroptotic pathway.

This proves that targeting ketolysis can help “hack” cancer cell resistance to ferroptosis through shutting down de novo lipogenesis and the HMGCR pathway.

SFAs, SFA-containing phospholipids (SFA-PLs), and cholesterol act as mechanical barriers for the propagation of lipid ROS, contributing to ferroptosis resistance. Inhibitors of enzymes involved in ketolysis and de novo lipogenesis, such as SCOT, ACC1, FASN, and SCD1, can reprogram the lipidome to make cancer cells more sensitive to ROS. Therefore, it is a promising approach to investigate the application of ferroptosis inducers in combination with inhibitors of de novo lipogenesis and ketolysis, especially for ferroptosis-resistant cancer types. The potential dependence of ferroptosis sensitivity on ketolysis inhibition has been demonstrated in this review for the first time. 

In general, increasing sensitivity to ferroptosis involves suppressing ketolysis and de novo lipogenesis, increasing the supply of PUFAs through nutrition, and promoting the accumulation of PUFAs in membrane phospholipids. Any metabolic reprogramming that decreases SFAs and MUFAs while increasing PUFAs in phospholipids holds promise for sensitizing cancer cells to ferroptosis. Combining activators of specific enzymes, such as phosphatidic acids for NOX activation, with ferroptosis inducers is also a possible strategy.

## Figures and Tables

**Table 1:** Oncogenes involved in de novo lipogenesis.

Oncogenes Regulate Lipid Metabolism	Metabolic and Lipidomic Changes	Reference
*ACC1*	synthesis of malonyl-CoA	[63]
*FASN*	synthesis of fatty acids	[67]
*SCD1*	desaturation of FA	[69]
*ELOVL6*	FA elongation	[71]

**Table 3:** Substances as ferroptosis inducers.

Small Molecules	Origin	Mechanism	Reference
erastin	synthetic	blocks cystine uptake which leads to depletion of glutathione	[1,4,5]
RSL3	synthetic	binds GPX4 and inhibits it	[6]
BSO	synthetic	depletes glutathione	[10]
sulfasalazine	synthetic	inhibits system xc-	[103]
sorafenib	synthetic	inhibits system xc-	[103]
artesunate	natural	elevates labile iron pool	[104]
dehydroartemisisnin	natural	decreases GSH level	[104]
withaferin A	natural	medium dose NRF2 activator; high dose GPX4 inhibitor	[106]
N6F11	synthetic	TRIM25 agonist, leads to degradation of GPX4	[30]

**Table 4:** Substances-inhibitors of de novo lipogenesis and ketolysis, prospective sensitizers to ferroptosis.

Target Protein or Pathway	Small Molecules	Origin	Perspective	Reference
ACC1 (i) *	soraphen A	natural	potential	[113]
ACC1 (i)	ND-646	synthetic	potential	[118]
FASN (i)	C75	synthetic	potential	[120]
FASN (i)	orlistat	natural	potential	[120]
FASN (i)	epigallocatechin-3-gallate	natural	potential	[120]
SCD1 (i)	CVT11127	synthetic	tested	[92]
SCD1 (i)	SSI-4	synthetic	potential	[121]
SCD1 (i)	aramchol (icomidocholic acid)	natural	potential	[70]
SCOT (i)	lithostat	synthetic	potential	[126,127]
SCOT-ACAT1 (i)	3- alpha R lipoic acid	natural	potential	[126,127]
SCOT-ACAT1 (i)	hydroxycitrate (from Garcinia Cambogia);	natural	potential	[126,127]
SCOT-ACAT1 (i) ACS (i)	allicin	natural	potential	[126,127]

* (i) inhibitor.

**Table 5:** Prospective sensitizers to ferroptosis.

Target Protein	Small Molecules	Origin	Perspective	Reference
PPARδ (a) */ACSL4	L-165041	synthetic	potential	[128]
LXR (a)/LPCAT3	saikosaponin A	natural	potential	[130]
LXR (a)/LPCAT3	24-hydroxycholesterol	natural	potential	[131]
CPT1a (i) **	etomoxir	synthetic	tested	[27]
CPT1 (i)	malonyl-CoA	synthetic	potential	[134,135]
HO-1 (a)	heme	synthetic	tested	[138]
HMGCR (i)	statins	synthetic	tested	[25]
HDAC (i)/Ferroportin	romidepsin	natural	tested	[140]

* (a) activator; ** (i) inhibitor.

**Table 6:** Reprogramming compounds focused on the activation of the proteins responsible for peroxidation.

Target Protein	Small Molecules	Origin	Perspective	Reference
NOX (a) *	Arachidonic acid (AA)	natural	potential	[141,146]
NOX (a)	Phosphatidic acids (PA)	natural	potential	[142]
NOX (a)	8,11,14-eicosatrienoic acid	natural	potential	[141]
ALOX15 (a)	PKUMDL_MH_1001	synthetic	tested	[80]
PEBP1 (a)/15LOX	locostatin	synthetic	tested	[24]

* (a) activator.
